# Mother’s Fruit Preferences and Consumption Support Similar Attitudes and Behaviors in Their Children

**DOI:** 10.3390/ijerph15122833

**Published:** 2018-12-12

**Authors:** Barbara Groele, Dominika Głąbska, Krystyna Gutkowska, Dominika Guzek

**Affiliations:** 1 Department of Dietetics, Faculty of Human Nutrition and Consumer Sciences, Warsaw University of Life Sciences (SGGW-WULS), 159C Nowoursynowska Street, 02-787 Warsaw, Poland; barbara_groele@sggw.pl; 2 Department of Organization and Consumption Economics, Faculty of Human Nutrition and Consumer Sciences, Warsaw University of Life Sciences (SGGW-WULS), 159C Nowoursynowska Street, 02-787 Warsaw, Poland; krystyna_gutkowska@sggw.pl (K.G.); dominika_guzek@sggw.pl (D.G.)

**Keywords:** fruit intake, fruit consumption behaviors, choice, preferences, mothers, children

## Abstract

Insufficient fruit intake is observed worldwide despite the generally higher preference for consumption of fruits than vegetables. For children, the determinants of consumption, such as at-home accessibility and parental consumption patterns, may especially influence fruit intake. The aim of this study was to analyze the association between fruit consumption behaviors and the preferences of mothers and their declared behaviors and preferences of children. The study was conducted in national samples of Polish (*n* = 1200) and Romanian (*n* = 1157) mothers of children aged 3–10 years (random quota sampling; quotas: age, education and place of residence) by using Computer-Assisted Telephone Interviewing (CATI). Mothers were asked about their general frequency of fruit consumption and their and their children’s most preferred fruits. A 24-h dietary recall of fruit intake was conducted for mothers and children. Significant associations were observed for (1) fruit consumption behaviors of mothers and children, (2) fruit consumption preferences of mothers and their declared preferences of their children, and (3) fruit consumption preferences of mothers and behaviors of their children. The associations were very strong for all fruits, both for Polish and Romanian samples. In order to increase the fruit intake of children, it is necessary to influence the fruit consumption preferences and behaviors of mothers.

## 1. Introduction

The World Health Organization (WHO) [1] indicates that fruit and vegetable intake is an essential element of diet that may contribute to the reduction of risk of noncommunicable diseases. Inadequate intake of fruit and vegetables has been estimated to contribute to around 6.7 million deaths in 2010 [2]. Several meta-analyses have shown the positive association between fruit or vegetable intake and health, and the recommended intake may reduce the risk of cardiovascular diseases [3], stroke [4], diabetes [5], hypertension [6], some types of cancers [4,7,8,9], and depression [10]. These beneficial effects result from the fiber content [11] or other bioactive compounds, characterized inter alia by antioxidative properties [12], but also from the lower energy value than other products [13]. Not only the quantity of consumed fruits and vegetables, but also their variety may positively influence health [14].

Considering the role of fruits and vegetables, the joint Food and Agricultural Organization (FAO) and WHO expert consultation formulated the recommendation of consuming at least 400 g of fruits and vegetables daily [15] and indicated it as a goal for all countries [16]. Despite the fact that a number of countries have implemented a policy of increasing fruit and vegetable intake [17], inadequate intake is a worldwide problem. Data from 52 countries participating in the World Health Survey indicated that 77.6% of men and 78.4% of women had a lower intake of fruits and vegetables than the minimum recommended level [18].

As the main reason for inadequate intake, the WHO [1] indicates the relatively high prices and limited access to fruits and vegetables when compared with other food products. However, for children, there are also additional determinants of fruit and vegetable intake that are associated with at-home accessibility [19], parental food consumption patterns [20], food neophobia [21], and general higher preferences for consumption of fruits than of vegetables [22]. Considering these aspects, it is necessary to analyze the role of mothers with their specific eating behaviors and preferences in influencing the eating behaviors and preferences of their children.

The aim of the study was to analyze the association between self-reported fruit consumption behaviors and preferences of mothers and their declared fruit consumption behaviors and preferences of their children in national samples of Polish and Romanian respondents.

## 2. Materials and Methods

### 2.1. Ethical Statement

The study was conducted in national samples of Polish and Romanian respondents according to the guidelines laid down in the Declaration of Helsinki. All procedures involving human subjects were approved by the ethics committee. Informed consent was provided by all participants.

### 2.2. Study Participants

The study was conducted in a national sample of 1200 Polish mothers of children aged 3–10 years and in a national sample of 1200 Romanian mothers of children aged 3–10 years, recruited using the random quota sampling method. The respondents were recruited by a professional international agency used for assessing public opinion and perception. The recruitment procedure was conducted using the random quota sampling method [23], with quotas for age, education, and place of residence (size of city and region).

The following inclusion criteria were applied: women, inhabitants of Poland (for Polish sample) or Romania (for Romanian sample), aged 25–45 years, mother of child/children aged 3–10 years, and who provided informed consent to participate in the study. The following exclusion criteria were applied: lack of registration for the audio material of informed consent to participate in the study; refused information about age, education, or place of residence (necessary for quota sampling); and missing data in the questionnaire.

For the Romanian sample, after the quota sampling procedure and obtaining informed consent to participate in the study, of the 1200 respondents, 43 had missing data in the questionnaire; therefore, the study was conducted in a national sample of 1157 Romanian mothers of children aged 3–10 years. For the Polish sample, after the quota sampling procedure and obtaining informed consent to participate in the study for 1200 respondents, there were no missing data in the questionnaires, so the study was conducted in a national sample of 1200 Polish mothers of children aged 3–10 years.

### 2.3. Study Design

The study was conducted using Computer-Assisted Telephone Interviewing (CATI) [24] by trained workers from the professional international agency used for assessing public opinion and perception. A questionnaire with open-ended questions about the fruit intake behaviors and preferences of mothers or their children was used. Before the interview, if the mother declared that she has more than one child aged 3–10 years, she was asked to answer about only one child and she was able to choose one child arbitrarily.

Mothers were asked about the general frequency of their fruit consumption (number of servings per day of raw and processed fruit) and their most preferred fruits (asked to list the most preferred). The 24-h dietary recall of fruit intake was then conducted, in which they were asked about the specific fruits consumed during the previous day with a specific serving size (the previous day consumption). They were also asked about the fruit consumption behaviors and preferences of their children—the general frequency of their fruit consumption (number of servings per day for raw and processed fruits) and the most preferred fruits (asked to list the most preferred). The 24-h dietary recall of fruit intake of children was then conducted, in which the mothers were asked about the specific fruits consumed by their children on the previous day with a specific serving size (the previous day consumption).

To avoid interruption while obtaining answers, there were some nonfactual “dummy” questions included between the indicated questions. The dummy questions were included in the questionnaire, even though they were not intended to be analyzed. They were associated with the interviewed issues of fruit consumption but were formulated in order to change the main area of questioning to prevent transfer of the answer from the previous question to the next one and to prevent the answers that followed from being influenced by the previous ones.

### 2.4. Statistical Analysis

For the statistical analysis, the differences between groups were verified using the chi-squared test. The distribution of data was verified using the Shapiro-Wilk test, and the analysis of correlation was then conducted based on Spearman’s rank correlation coefficient due to the nonparametric distribution of data.

The level of significance was set as *p* ≤ 0.05. Statistical analysis was carried out using the Statgraphics Plus for Windows 5.1 (Statgraphics Technologies Inc., The Plains, VA, USA) and the Statistica software version 8.0 (StatSoft Inc., Tulsa, OK, USA).

## 3. Results

The analysis of the association between the fruit consumption behaviors of mothers and their declared fruit consumption behaviors of their children in a national sample of Polish respondents is presented in Table 1. We observed that, in the Polish population for all the declared fruits, despite various shares of consumers (18.2–83.6% of children consuming when mother consumes), the consumption behaviors of mothers and children were significantly associated. The percentage of children consuming differed significantly between groups (*p* < 0.0001).

The analysis of the association between the fruit consumption behaviors of mothers and their declared fruit consumption behaviors of their children in a national sample of Romanian respondents is presented in Table 2. We observed that, in the Romanian population for all the declared fruits, the consumption behaviors of mothers and children were significantly associated. The percentage of children consuming differed significantly between groups (*p* < 0.0001).

The analysis of the association between the fruit preferences of mothers and their declared fruit preferences of their children in a national sample of Polish respondents is presented in Table 3. We observed that in the Polish population for all the declared fruits, despite the range in consumption (17.5–70.9% of children preferring when mother prefers), the preferences of mothers and their declared preferences of their children were significantly associated. The percentage of children preferring fruit differed significantly between groups (*p* < 0.0001).

The analysis of the association between the fruit preferences of mothers and their declared fruit preferences of their children in a national sample of Romanian respondents is presented in Table 4. We observed that in the Romanian population for all the declared fruits, despite the range of consumers (4.4–70.0% of children preferring when mother prefers), the preferences of mothers and their declared preferences of their children were significantly associated. The percentage of children preferring differed significantly between groups (*p* < 0.01).

The analysis of the association between the fruit preferences of mothers and their declared fruit consumption behaviors of their children in a national sample of Polish respondents is presented in Table 5. We observed that, in the Polish population for all the declared fruits, despite the range in consumption (3.8–74.8% of children consuming when mother prefers), the preferences of mothers and their declared consumption behaviors of their children were significantly associated. The percentage of children consuming differed significantly between groups (*p* < 0.05).

The analysis of the association between the fruit preferences of mothers and their declared fruit consumption behaviors of their children in a national sample of Romanian respondents is presented in Table 6. We observed that, in the Romanian population for the majority of declared fruits, despite the range in consumption (4.4–64.5% of children consuming when mother prefers), the preferences of mothers and their declared consumption behaviors of their children were significantly associated. The percentage of children consuming differed significantly between groups (*p* < 0.05), except for strawberries (*p* = 0.6088).

The analysis of the correlation between the number of servings of fruit consumed per day by mothers and their declared consumption of their children in a national sample of Polish respondents is presented in Table 7. The median of declared general number of servings of fruit consumed per day for both mothers and children was two, and it varied from no servings (consumed less often than once a week) to five servings (nonparametric distribution). The median declared number of servings of fruit consumed on the previous day for both mothers and children was two, ranging from no servings at all to 10 servings (for children) and 15 servings (for mothers) (nonparametric distribution). We observed that, in the Polish population both for general number of servings (assessed on the basis of the individual declaration of fruit intake) and previous day number of servings (assessed based on the 24-h dietary recall of fruit intake), the intake of mothers and children was significantly associated (*p* < 0.0001).

The analysis of the correlation between the number of servings of fruit consumed per day by mothers, and their declared consumption of their children in a national sample of Romanian respondents is presented in Table 8. The median of declared general number of servings of fruit consumed per day for both mothers and children was two, ranging from no servings (consumed less often than once a week) to five servings (nonparametric distribution). The median of declared number of servings of fruit consumed on the previous day for both mothers and children was two, ranging from no servings at all to 10 servings (for children) and 14 servings (for mothers) (nonparametric distribution). We observed in the Romanian population, both for general number of servings (assessed on the basis of individual declaration of fruit intake) and previous day number of servings (assessed on the basis of the 24-h dietary recall of fruit intake), that the intake of mothers and children was significantly associated (*p* < 0.0001).

## 4. Discussion

The association between parental diet quality and the characteristics of the children’s diet has been observed in a number of studies. Despite findings of a moderate or weak association, Wang et al. [25] confirmed this association in their systematic review and meta-analysis. In the systematic review and meta-analysis of Yee et al. [26], this association was attributed to various mechanisms of active guidance or education, psychosocial mediators, and moderating influence of general parenting styles.

Specifically, for fruit and vegetable intake, in a systematic review by Pearson et al. [27], general parental fruit and vegetable intake was found to be consistently positively associated with childrens’ and adolescents’ fruit and vegetable intake. However, this association between parental fruit and vegetable dietary habits and relevant dietary habits of children was sometimes not observed, even if it was found for the general diet quality or for other products. For example, Davison et al. [28] reported that, for 9- to 11-year-old children, higher parental diet quality was associated with lower consumption of confectionery, chocolate, cakes, biscuits, and savory snacks in children, with no association for fruits and vegetables, but such results are rather rare.

In the present study, a deeper analysis of fruit intake was conducted, as not only a general association was observed (for the number of servings), but also specific associations were investigated (for the consumption of specific fruits). Both declared diet and declared preferences were analyzed. The type of consumed and preferred fruits may be a crucial issue, as it has been proven that experiencing various tastes and flavors in early life promotes a healthy diet and influences a wider consumption of fruits and vegetables [29]. Therefore, not only the number of serving of fruits consumed by the child is important, but exposure to various fruits is also important to enable future consumption and preferences.

The results of the present study conducted in two countries characterized by diverse national fruit supply—according to the Statistics Division of the FAO (FAO STAT): 60.17 kg per capita per year for Poland and 76.17 kg per capita per year for Romania [30]—indicate similar observations. A large share of respondents declared neither consumption nor preferences for a number of fruits, whereas only apples were declared as consumed and preferred by a majority of mothers in both countries. This indicates the potential risk of insufficient early life exposure, resulting in children having low consumption and preference for a number of fruits.

In conclusion, if a mother does not consume a specific fruit, her child does not consume this specific type of fruit. We observed in both the Polish and Romanian sample that the proportion of children consuming specific fruits differed significantly between groups stratified by the mother’s eating habits. However, if not preferences but habits (assessed in the present study on the basis of the previous day dietary recall) are interpreted, additional influencing physical factors may include at-home availability and accessibility [27]. If both mothers and children did not consume a number of fruits (only apples were declared as consumed by a majority of respondents), the consistent non-consumption of mothers and children was confirmed by a higher frequency of non-consuming children if the mother did not consume the fruit. This finding may have resulted from a lack of at-home availability and accessibility. This may be a problem especially for children with a low general fruit preference, as for children characterized by low fruit or vegetable preferences, in order to increase their intake, at-home availability and accessibility are necessary. Thus, parents must make efforts to provide fruit and inform their child about availability [31]. In a group assessed in this study, such need may be emphasized as, for the majority of children, many fruits were stated to be non-preferred, when their mother expressed similar dislike, suggesting that these foods were not available or accessible to the child.

The other important observation, which was consistent for both Poland and Romania, was that the mother’s own preference was declared by her as the preference of her child. Such similar preferences result from the general at-home preference learning, which is based on the transmission of cultural and familial beliefs, attitudes, and practices, associated with what, when, and how much to eat [32]. Children are aware of their parents’ nutritional eating habits and deliberately adopt those habits [20]. However, this is not the only explanation for the consistent preferences declared by mothers for themselves and their children. The other reason is reporter bias, in which the reported diet of a child resembles the diet of the parent who provides information about it, despite the fact that the general association between the diet of mothers and the diet of children is stronger than that between the diet of fathers and the diet of children. As such, reporter bias may also be an influencing factor [33]. In terms of this approach, our observations may be explained by the fact that a mother who does not prefer a specific fruit does not provide accessibility to that fruit, as she believes that her child also dislikes it. This is confirmed by the fact that, in the present study, the preference of the mother was significantly associated with fruit consumption behaviors of their children in both Polish and Romanian samples. This mechanism is a serious factor that may interfere with at-home learning of preference and, as a result, reduce the intake of specific fruits.

For children, a number of factors influence their intake: the general factors observed for all consumers (e.g., individual preferences), specific factors associated with their parents (e.g., family meals), and specific factors associated with the strong influence of media and peers (e.g., television commercials) [29]. As a result, it is commonly observed that the quality of a child’s diet is lower than that of their parents [34]. In Western communities, children overestimate their fruit intake [35] and they easily adopt the adverse nutritional habits of their parents [36], as the non-recommended health-related behaviors are easier and more attractive than recommended behaviors [37]. Consequently, a child accumulates the adverse nutritional habits of parents and peers, sometimes with almost no chance to change due to a lack of at-home exposure to recommended products.

It is necessary to overcome the unfavorable situation created when a mother does not prefer a certain fruit and does not purchase it for her family. A lack of exposure leads to a lack of preference by her children. Therefore, choice for children must be provided. A mother who does not like a specific fruit probably will not consume it, but she should at least give her child an opportunity to try it. This need is associated with the fact that at-home exposure of children results not only in higher preferences but also higher intake—both at home and at school lunch [38]. In a Polish nationwide sample, an association between higher fruit and vegetable intake, higher variety of product exposure, lower food neophobia level, more frequent socializing with peers, and more frequent physical activity confirmed that all of these factors aid in the development of proper nutritional habits [39]. Therefore, mothers must be educated about the need for exposure, as nutritional knowledge of parents is indicated to be crucial for influencing the fruit and vegetable consumption of their children [40].

In order to increase fruit consumption in children, it is necessary to educate parents with regard to the fruit intake recommended for children and the fruit intake recommended for them (about quantity and variability). The nutritional behaviors of children and their preferences are created as a result of nutritional behaviors and preferences of their parents; therefore, their preferences and behaviors may be changed later as a result of changing preferences and behaviors of their parents. This study confirms previous observations that interventions to increase fruit and vegetable intake of children must target parents’ own intake [41]. However, the observations from this study conducted in national Polish and Romanian samples indicate that such association exists for the general intake and for specific fruits; hence, diversity may also be influenced and must be supported by at-home accessibility.

## 5. Conclusions

In conclusion, the non-consumption of a specific fruit by mothers in national Polish and Romanian samples was accompanied by non-consumption of the same fruit by their children. This may have resulted from a lack of at-home availability and accessibility. The lack of preference for specific fruits by mothers may result in lack of at-home exposure of children and a subsequent lack of preference in their children. The fruit preferences declared by mothers were consistent with those declared by them for their children. This may have resulted from general at-home learning of food preferences as well as reporter bias, as the reporting parent perceives the preferences of their child as resembling their own.

There is a need to educate women about the necessity to expose their children in early childhood to various types of fruits even if a specific fruit is not preferred by mothers in order to avoid limiting the choice of fruits consumed by their children and to create beneficial dietary habits. The recommendation to consume a variety of fruits every day should be taught to parents as a guideline for both children and parents, because if a mother does not consume fruit, her child follows the same behavior.

## Figures and Tables

**Table 1:** Analysis of the association between the fruit consumption behaviors of mothers and their declared fruit consumption behaviors of their children in a national sample of Polish respondents (*n* = 1200).

Fruit	Mothers Consuming the Specified Fruit ^b^		Mothers Not Consuming the Specified Fruit ^b^		*p*-Value ^c^
	Declaring Their Children as Also Consuming	Declaring Their Children as Not Consuming	Declaring Their Children as Consuming	Declaring Their Children as Also Not Consuming	
Apple (*n* = 843; *n* = 357) ^a^	705 (83.6%)	138 (16.4%)	158 (44.3%)	199 (55.7%)	<0.0001
Banana (*n* = 421; *n* = 779) ^a^	298 (70.8%)	123 (29.2%)	226 (29.0%)	553 (71.0%)	<0.0001
Tangerine (*n* = 280; *n* = 920) ^a^	177 (63.2%)	103 (36.8%)	92 (10.0%)	828 (90.0%)	<0.0001
Orange (*n* = 145; *n* = 1055) ^a^	61 (42.1%)	84 (57.9%)	50 (4.7%)	1005 (95.3%)	<0.0001
Grapes (*n* = 122; *n* = 1078) ^a^	68 (55.7%)	54 (44.3%)	55 (5.1%)	1023 (94.9%)	<0.0001
Pear (*n* = 108; *n* = 1092) ^a^	63 (58.3%)	45 (41.7%)	59 (5.4%)	1033 (94.6%)	<0.0001
Plum (*n* = 104; *n* = 1096) ^a^	57 (54.8%)	47 (45.2%)	37 (3.4%)	1059 (96.6%)	<0.0001
Peach (*n* = 54; *n* = 1146) ^a^	27 (50.0%)	27 (50.0%)	23 (2.0%)	1123 (98.0%)	<0.0001
Grapefruit (*n* = 33; *n* = 1167) ^a^	6 (18.2%)	27 (81.8%)	8 (0.7%)	1159 (99.3%)	<0.0001
Strawberry (*n* = 39; *n* = 1161) ^a^	18 (46.2%)	21 (53.8%)	32 (2.8%)	1129 (97.2%)	<0.0001
Watermelon (*n* = 12; *n* = 1188) ^a^	7 (58.3%)	5 (41.7%)	4 (0.3%)	1184 (99.7%)	<0.0001

^a^ The number of mothers consuming the specific fruit followed by the number of mothers not consuming the specific fruit; ^b^ Assessed on the basis of the previous day fruit consumption behaviors (24-h dietary recall); ^c^ Chi^2^ test.

**Table 2:** Analysis of the association between the fruit consumption behaviors of mothers and their declared fruit consumption behaviors of their children in a national sample of Romanian respondents (*n* = 1157).

Fruit	Mothers Consuming the Specified Fruit ^b^		Mothers Not Consuming the Specified Fruit ^b^		*p*-Value ^c^
	Declaring Their Children as Also Consuming	Declaring Their Children as Not Consuming	Declaring Their Children as Consuming	Declaring Their Children as Also Not Consuming	
Apple (*n* = 677; *n* = 480) ^a^	498 (73.6%)	179 (26.4%)	177 (36.9%)	303 (63.1%)	<0.0001
Banana (*n* = 329; *n* = 828) ^a^	236 (71.7%)	93 (28.3%)	231 (27.9%)	597 (72.1%)	<0.0001
Orange (*n* = 263; *n* = 894) ^a^	176 (66.9%)	87 (33.1%)	92 (10.3%)	802 (89.7%)	<0.0001
Tangerine (*n* = 184; *n* = 973) ^a^	121 (65.8%)	63 (34.2%)	62 (6.4%)	911 (93.6%)	<0.0001
Grapes (*n* = 140; *n* = 1017) ^a^	88 (62.9%)	52 (37.1%)	55 (5.4%)	962 (94.6%)	<0.0001
Pear (*n* = 96; *n* = 1061) ^a^	51 (53.1%)	45 (46.9%)	50 (4.7%)	1011 (95.3%)	<0.0001
Peach (*n* = 93; *n* = 1064) ^a^	52 (55.9%)	41 (44.1%)	30 (2.8%)	1034 (97.2%)	<0.0001
Plum (*n* = 85; *n* = 1072) ^a^	50 (58.8%)	35 (41.2%)	36 (3.4%)	1036 (96.9%)	<0.0001
Watermelon (*n* = 41; *n* = 1116) ^a^	23 (56.1%%)	18 (43.9%)	11 (1.0%)	1105 (99.0%)	<0.0001
Grapefruit (*n* = 15; *n* = 1142) ^a^	6 (40.0%)	9 (60.0%)	5 (0.4%)	1137 (99.6%)	<0.0001
Strawberry (*n* = 5; *n* = 1152) ^a^	2 (40.0%)	3 (60.0%)	5 (0.4%)	1147 (99.6%)	<0.0001

^a^ The number of mothers consuming the specific fruit followed by the number of mothers not consuming the specific fruit; ^b^ assessed on the basis of the previous day fruit consumption behaviors (24-h dietary recall); ^c^ Chi^2^ test.

**Table 3:** Analysis of the association between the fruit preferences of mothers and their declared fruit preferences of their children in a national sample of Polish respondents (*n* = 1200).

Fruit	Mothers Indicating the Specified Fruit as the most Preferred		Mothers Not Indicating the Specified Fruit as the most Preferred		*p*-Value ^b^
	Declaring Their Children as Also Preferring	Declaring Their Children as Not Preferring	Declaring Their Children as Preferring	Declaring Their Children as Also Not Preferring	
Apple (*n* = 721; *n* = 479) ^a^	511 (70.9%)	210 (29.1%)	255 (53.2%)	224 (46.8%)	<0.0001
Banana (*n* = 419; *n* = 781) ^a^	292 (69.7%)	127 (30.3%)	386 (49.4%)	395 (50.6%)	<0.0001
Tangerine (*n* = 256; *n* = 944) ^a^	129 (50.4%)	127 (49.6%)	198 (21.0%)	746 (79.0%)	<0.0001
Strawberry (*n* = 227; *n* = 973) ^a^	107 (47.1%)	120 (52.9%)	152 (15.6%)	821 (84.4%)	<0.0001
Grapes (*n* = 224; *n* = 976) ^a^	97 (43.3%)	127 (56.7%)	116 (11.9%)	860 (88.1%)	<0.0001
Pear (*n* = 190; *n* = 1010) ^a^	75 (39.5%)	115 (60.5%)	126 (12.5%)	884 (87.5%)	<0.0001
Orange (*n* = 170; *n* = 1030) ^a^	55 (32.4%)	115 (67.6%)	91 (8.8%)	939 (91.2%)	<0.0001
Watermelon (*n* = 105; *n* = 1095) ^a^	46 (43.8%)	59 (56.2%)	46 (4.2%)	1049 (95.8%)	<0.0001
Plum (*n* = 101; *n* = 1099) ^a^	19 (18.8%)	82 (81.2%)	57 (5.2%)	1042 (94.8%)	<0.0001
Peach (*n* = 92; *n* = 1108) ^a^	19 (20.7%)	73 (79.3%)	57 (5.1%)	1051 (94.9%)	<0.0001
Grapefruit (*n* = 57; *n* = 1143) ^a^	10 (17.5%)	47 (82.5%)	15 (1.3%)	1128 (98.7%)	<0.0001

^a^ The number of mothers indicating the specific fruit as the most preferred followed by the number of mothers not indicating the specific fruit as the most preferred; ^b^ Chi^2^ test.

**Table 4:** Analysis of the association between the fruit preferences of mothers and their declared fruit preferences of their children in a national sample of Romanian respondents (*n* = 1157).

Fruit	Mothers Indicating the Specified Fruit as the Most Preferred		Mothers Not Indicating the Specified Fruit as the Most Preferred		*p*-Value ^b^
	Declaring Their Children as Also Preferring	Declaring Their Children as Not Preferring	Declaring Their Children as Preferring	Declaring Their Children as Also Not Preferring	
Apple (*n* = 636; *n* = 521) ^a^	380 (59.7%)	256 (40.3%)	196 (37.6%)	325 (62.4%)	<0.0001
Banana (*n* = 347; *n* = 810) ^a^	243 (70.0%)	104 (20.0%)	389 (48.0%)	421 (52.0%)	<0.0001
Orange (*n* = 306; *n* = 851) ^a^	132 (43.1%)	174 (56.9%)	186 (21.9%)	665 (78.1%)	<0.0001
Grapes (*n* = 293; *n* = 864) ^a^	105 (35.8%)	188 (64.2%)	120 (13.9%)	744 (86.1%)	<0.0001
Pear (*n* = 177; *n* = 980) ^a^	62 (35.0%)	115 (65.0%)	97 (9.9%)	883 (90.1%)	<0.0001
Peach (*n* = 168; *n* = 989) ^a^	48 (28.6%)	120 (71.4%)	60 (6.1%)	929 (93.9%)	<0.0001
Tangerine (*n* = 128; *n* = 1029) ^a^	52 (40.6%)	76 (59.4%)	117 (11.4%)	912 (88.6%)	<0.0001
Watermelon (*n* = 120; *n* = 1037) ^a^	58 (47.9%)	63 (52.1%)	65 (6.3%)	972 (93.7%)	<0.0001
Strawberry (*n* = 102; *n* = 1055) ^a^	27 (26.5%)	75 (73.5%)	97 (9.2%)	958 (90.8%)	<0.0001
Plum (*n* = 73; *n* = 1084) ^a^	15 (20.5%)	58 (79.5%)	97 (8.9%)	987 (91.1%)	<0.0001
Grapefruit (*n* = 45; *n* = 1112) ^a^	2 (4.4%)	43 (95.6%)	8 (0.7%)	1104 (99.3%)	0.0081

^a^ The number of mothers indicating the specific fruit as the most preferred followed by the number of mothers not indicating the specific fruit as the most preferred; ^b^ Chi^2^ test.

**Table 5:** Analysis of the association between the fruit preferences of mothers and their declared fruit consumption behaviors of their children in a national sample of Polish respondents (*n* = 1200).

Fruit	Mothers Indicating the Specified Fruit as the Most Preferred		Mothers Not Indicating the Specified Fruit as the Most Preferred		*p*-Value ^b^
	Declaring Their Children as Consuming	Declaring Their Children as Not Consuming	Declaring Their Children as Consuming	Declaring Their Children as Not Consuming	
Apple (*n* = 721; *n* = 479) ^a^	539 (74.8%)	182 (25.2%)	324 (67.6%)	155 (32.4%)	0.0072
Banana (*n* = 419; *n* = 781) ^a^	203 (48.4%)	216 (51.6%)	321 (41.1%)	460 (58.9%)	0.0144
Tangerine (*n* =2 56; *n* = 944) ^a^	108 (42.2%)	148 (57.8%)	161 (17.1%)	783 (82.9%)	<0.0001
Strawberry (*n* = 227; *n* = 973) ^a^	18 (7.9%)	209 (92.1%)	32 (3.3%)	941 (96.7%)	0.0016
Grapes (*n* = 224; *n* = 976) ^a^	51 (22.8%)	173 (77.2%)	72 (7.4%)	904 (92.6%)	<0.0001
Pear (*n* = 190; *n* = 1010) ^a^	40 (21.1%)	150 (78.9%)	82 (8.1%)	928 (91.9%)	<0.0001
Orange (*n* = 170; *n* = 1030) ^a^	34 (20.0%)	136 (80.0%)	77 (7.5%)	953 (92.5%)	<0.0001
Watermelon (*n* = 105; *n* = 1095) ^a^	4 (3.8%)	101 (96.2%)	8 (0.7%)	1087 (99.3%)	0.0025
Plum (*n* = 101; *n* = 1099) ^a^	22 (21.8%)	79 (78.2%)	72 (6.6%)	1027 (93.4%)	<0.0001
Peach (*n* = 92; *n* = 1108) ^a^	10 (10.9%)	82 (89.1%)	40 (3.6%)	1068 (96.4%)	0.0009
Grapefruit (*n* = 57; *n* = 1143) ^a^	4 (7.0%)	53 (93.0%)	10 (0.9%)	1133 (99.1%)	<0.0001

^a^ The number of mothers indicating the specific fruit as the most preferred followed by the number of mothers not indicating the specific fruit as the most preferred; ^b^ Chi^2^ test.

**Table 6:** Analysis of the association between the fruit preferences of mothers and their declared fruit consumption behaviors of their children in a national sample of Romanian respondents (*n* = 1157).

Fruit	Mothers Indicating the Specified Fruit as the Most Preferred		Mothers Not Indicating the Specified Fruit as the Most Preferred		*p*-Value ^b^
	Declaring Their Children as Consuming	Declaring Their Children as Not Consuming	Declaring Their Children as Consuming	Declaring Their Children as Not Consuming	
Apple (*n* = 636; *n* = 521) ^a^	410 (64.5%)	226 (35.5%)	265 (50.9%)	256 (49.1%)	<0.0001
Banana (*n* = 347; *n* = 810) ^a^	153 (44.1%)	194 (55.9%)	314 (38.8%)	496 (61.2%)	<0.0001
Orange (*n* = 306; *n* = 851) ^a^	111 (36.3%)	195 (63.7%)	157 (18.4%)	694 (81.6%)	<0.0001
Grapes (*n* = 293; *n* = 864) ^a^	65 (22.2%)	228 (77.8%)	78 (9.0%)	786 (91.0%)	<0.0001
Pear (*n* = 177; *n* = 980) ^a^	32 (18.1%)	145 (81.9%)	69 (7.0%)	911 (93.0%)	<0.0001
Peach (*n* = 168; *n* = 989) ^a^	28 (16.7%)	140 (83.3%)	54 (5.5%)	935 (94.5%)	<0.0001
Tangerine (*n* = 128; *n* = 1029) ^a^	55 (43.0%)	73 (57.0%)	128 (12.4%)	901 (87.6%)	<0.0001
Watermelon (*n* = 120; *n* = 1037) ^a^	16 (13.3%)	104 (86.7%)	18 (1.7%)	1019 (98.3%)	<0.0001
Strawberry (*n* = 102; *n* = 1055) ^a^	1 (1.0%)	101 (99.0%)	6 (0.6%)	1049 (99.4%)	0.6088
Plum (*n* = 73; *n* = 1084) ^a^	14 (19.2%)	59 (80.8%)	72 (6.6%)	1012 (93.4%)	0.0001
Grapefruit (*n* = 45; *n* = 1112) ^a^	2 (4.4%)	43 (95.6%)	9 (0.8%)	1103 (99.2%)	0.0138

^a^ The number of mothers indicating the specific fruit as the most preferred followed by the number of mothers not indicating the specific fruit as the most preferred; ^b^ Chi^2^ test.

**Table 7:** Analysis of the correlation between the number of servings of fruit consumed per day by mothers, and their declared consumption by their children in a national sample of Polish respondents (*n* = 1200).

Variable		Declared Number of Servings of Fruit Consumed Per Day by Mother			
		General Number of Servings ^a^		Previous Day Number of Servings ^b^	
		*p*-Value ^c^	R	*p*-Value ^c^	R
Declared by mother number of servings of fruit consumed per day by child	General number of servings ^a^	<0.0001	0.5326	<0.0001	0.4221
	Previous day number of servings ^b^	<0.0001	0.4122	<0.0001	0.5277

^a^ Assessed on the basis of the individual declaration of fruit intake; ^b^ assessed on the basis of the 24-h dietary recall of fruit intake; ^c^ the Spearman’s rank correlation coefficient.

**Table 8:** Analysis of the correlation between the number of servings of fruit consumed per day by mothers, and their declared consumption by their children in a national sample of Romanian respondents (*n* = 1157).

Variable		Declared Number of Servings of Fruit Consumed Per Day by Mother			
		General Number of Servings ^a^		Previous Day Number of Servings ^b^	
		*p*-Value ^c^	R	*p*-Value ^c^	R
Declared by mother number of servings of fruit consumed per day by child	General number of servings ^a^	<0.0001	0.5349	<0.0001	0.4158
	Previous day number of servings ^b^	<0.0001	0.4006	<0.0001	0.4732

^a^ Assessed on the basis of the individual declaration of fruit intake; ^b^ assessed on the basis of the 24-h dietary recall of fruit intake; ^c^ the Spearman’s rank correlation coefficient.
